# Application of multi-channel magnetic particle immunofluorescent disc microfluidic chip for combined detection of antibodies against six common infectious diseases including visceral leishmaniasis in pastoral areas

**DOI:** 10.3389/fcimb.2026.1693458

**Published:** 2026-03-10

**Authors:** Yiran Wang, Junhao Li

**Affiliations:** 1 School of Clinical Medicine, Xinjiang Second Medical College, Karamay, China; 2 School of Public Health, Xinjiang Second Medical College, Karamay, China

**Keywords:** combined detection, infectious disease antibodies, magnetic particle immunofluorescence, multi-channel microfluidic chip, pastoral areas

## Abstract

**Objectives:**

This study aimed to develop a microfluidic chip for the simultaneous detection of specific antibodies against six common pathogens in pastoral areas. The specific detection targets include: visceral leishmaniasis (IgG against *Leishmania* soluble antigen, IgG against recombinant K39 antigen); cystic echinococcosis (IgG against *Echinococcus* granulosus antigen 5, IgG against *Echinococcus* granulosus antigen B); alveolar echinococcosis (IgG against *Echinococcus* multilocularis antigen 2, IgG against *Echinococcus* multilocularis antigen 18); brucellosis (IgG against *Brucella* lipopolysaccharide, IgM against *Brucella* lipopolysaccharide); Lyme disease (IgG against *Borrelia* burgdorferi, IgM against *Borrelia* burgdorferi); and Xinjiang hemorrhagic fever (virus-specific IgG, virus-specific IgM).

**Methods:**

Based on magnetic particle immunofluorescence, a multi-channel disc microfluidic chip and reagents were designed. Parameters (antigen-antibody microsphere ratio, sample dilution) were optimized; fluid dynamics tests verified fluid operation feasibility. Serum samples from patients with the six diseases and healthy controls were used to detect target antibodies. The chip’s accuracy, dose-response curve (R^2^), limit of detection (LOD), precision, and specificity were evaluated. Bland-Altman analysis compared results with traditional ELISA to assess consistency.

**Results:**

The chip exhibited normal fluidic operation. Optimized reagents/samples enhanced utilization efficiency. For the six diseases, the detection accuracy met the requirements: the coefficient of determination (R^2^) for all indicators was greater than 0.98, the minimum limit of detection (LOD) among the 12 detection items was 0.6 μg/mL, the relative standard deviation (RSD) for precision was less than 10%, and no cross-reactions occurred. Bland-Altman consistency analysis showed that the differences in detection results met clinically acceptable standards, demonstrating good consistency with enzyme-linked immunosorbent assay (ELISA).

**Conclusions:**

This chip exhibits stable fluid flow, low sample/reagent consumption, and excellent accuracy/precision/specificity. Its detection results are consistent with those of traditional methods, and it enables the simultaneous combined detection of the aforementioned six infectious diseases. Compared with the clinically used method (enzyme-linked immunosorbent assay, ELISA), this chip has greater advantages in application scenarios and comprehensive benefits; compared with existing disc-based microfluidic technologies, it holds superior advantages in terms of detection throughput and application objectives. It provides a rapid, sensitive, and specific technical tool for the acute-phase screening, disease course confirmation, infection staging, differentiation between bacterial and parasitic infections, and disease prognosis detection of multiple prevalent infectious diseases in pastoral areas.

## 1 Introduction

As globally important livestock husbandry regions, pastoral areas exhibit unique geographical features, climatic characteristics, and distinct local residents’ production and living patterns. However, this uniqueness—including vast grazing lands, close human-animal cohabitation environments, and favorable conditions for the natural reproduction of vectors such as ticks and sandflies—also imposes significant pressure on infectious disease prevention and control (Liu et al., 2022; Kui et al., 2024). Among various infectious diseases, six are highly prevalent in pastoral areas worldwide: visceral leishmaniasis (kala-azar), echinococcosis (encompassing cystic and alveolar forms), brucellosis (commonly abbreviated as brucellosis), Lyme disease, and Xinjiang hemorrhagic fever (also known as Crimean-Congo hemorrhagic fever). Specifically, visceral leishmaniasis is endemic in sub-Saharan African pastoral areas, the Asian Indian subcontinent, Central Asian steppes, and the northeastern Brazilian livestock belt (Safavi et al., 2021). Caused by *Leishmania* parasite infection, it results in approximately 500,000 new cases annually, with 350 million people at risk of infection. Without standardized treatment, over 95% of patients die from complications (Desjeux, 2024). Additionally, medical expenses incurred by patients place a heavy financial burden on pastoral families (Abebe et al., 2024).Regarding echinococcosis, cystic echinococcosis is widely distributed in European Mediterranean pastoral areas, Australian (Oceania) sheep-farming regions, and the Argentine Pampas (South America), while alveolar echinococcosis is concentrated in the European Alps (Northern Hemisphere), Asian Mongolian steppes, and western North American pastoral areas (Lupia et al., 2021). Echinococcosis is caused by the larvae of *Echinococcus* tapeworms parasitizing the human body: lesions of cystic echinococcosis may rupture suddenly, triggering anaphylactic shock and even death; if alveolar echinococcosis is not treated promptly, its 10-year mortality rate exceeds 90%, earning it the nickname “parasitic cancer.” In some pastoral villages severely affected by the epidemic, echinococcosis reduces livestock production by approximately 15%–20% annually, leading to economic losses of over several million yuan (RMB) (Weber et al., 2023; Jensenius et al., 2024).Brucellosis, caused by *Brucella* infection, is one of the most common zoonoses globally and is highly endemic in European (Spanish, Italian) cattle-sheep producing areas, African (Nigerian, Kenyan) nomadic tribes, and Asian (Iranian, Mongolian) grasslands (Raad, 2021). Severe cases involve joints and internal organs, leaving long-term sequelae and even life-threatening complications. Meanwhile, infected livestock must be culled, and additional investments are required for farm disinfection and epidemic prevention measures, inflicting severe damage on the pastoral economy (Qureshi et al., 2023). Lyme disease, caused by *Borrelia* burgdorferi infection, has an endemic range that highly overlaps with tick habitats in pastoral areas: herdsmen in northeastern North America’s New England region, central European (German) Bavarian grasslands, and Asian (northeastern China, Siberian Russia) pastoral areas are at high risk due to frequent contact with tick hosts (deer, rodents) (Robert, 2025). As the disease progresses, it affects multiple organs such as the nervous and cardiovascular systems, potentially leading to severe consequences or even death. Families of some severe cases in pastoral areas have fallen into poverty due to medical expenses (Mead, 2022).Xinjiang hemorrhagic fever, caused by the Xinjiang hemorrhagic fever virus, is the only tick-borne hemorrhagic fever endemic across Europe, Asia, and Africa. In addition to China’s Xinjiang pastoral areas, it is also highly endemic in African (Sudanese, Ethiopian) livestock areas and Middle Eastern (Iranian, Iraqi) grasslands; cattle and sheep serve as both tick hosts and key virus reservoirs (Wang et al., 2024). It is characterized by acute onset, severe symptoms, and high mortality. Due to its sporadic nature, prevention and control are challenging, requiring substantial human and material resources for monitoring and early warning. Furthermore, the cost of emergency treatment for patients after onset is exorbitant (Kong et al., 2022). Collectively, these diseases not only seriously threaten the health of pastoral residents but also cause enormous local economic losses (Ghosh et al., 2021; Alvi et al., 2023).

Currently, early screening for these infectious diseases still primarily relies on traditional serological testing, among which the enzyme-linked immunosorbent assay (ELISA) is the most widely used. ELISA demonstrates good performance in terms of sensitivity and specificity (Mahdis and Marya, 2022). However, this detection method still has several limitations, such as relatively long detection time, complex operational procedures, high consumption of samples and reagents, and inability to efficiently achieve simultaneous detection of multiple diseases (Kauter et al., 2023). When using ELISA to measure the level of a novel infliximab, the Delgado research team compared it with rapid point-of-care testing (POCT). The results showed that ELISA not only requires a relatively long detection time (ranging from several hours to one day) but also relies on laboratory equipment; this finding further confirms the issue of “low detection efficiency” associated with ELISA (Teresa et al., 2024). Moreover, medical resources in pastoral areas are more scarce compared to urban areas, which exacerbates the aforementioned limitations of ELISA. For instance, timely testing is unavailable in some regions, increasing the difficulty of early screening and prompt prevention/control of these diseases, which in turn leads to delayed treatment, increased treatment complexity, and elevated risk of disease transmission (Legesse et al., 2023).

As an emerging category of detection technology, microfluidic chip technology possesses prominent advantages such as integration, miniaturization, and automation. In recent years, microfluidic technology has gradually demonstrated technical advantages in rapid sample processing capabilities and simultaneous detection of multiple indicators, showing broad application prospects in the field of timely detection of infectious diseases (Sun et al., 2022). Multiple studies have provided support for this: the Jamiruddin team summarized the specific applications of microfluidic technology in the detection of SARS-CoV-2 nucleic acids, antigens, and antibodies through systematic research (Mohd et al., 2022); Yang focused his research on microfluidic point-of-care (POCT) devices, exploring their application value in the early diagnosis of infectious diseases, tumors, and chronic diseases. Meanwhile, he analyzed the integration potential of these devices with wearable devices and telemedicine, and proposed feasible solutions for the popularization of this technology in resource-scarce areas (Yang et al., 2022); the Ayesha team successfully developed a paper-based microfluidic device (μPAD). This device combines dendrimer signal amplification technology with mass spectrometry detection methods, enabling the detection of Plasmodium falciparum antigens with only 30 μL of whole blood. Furthermore, verification results in asymptomatic populations in Ghana showed that its sensitivity reached as high as 96.5%, making it highly suitable for primary screening scenarios (Ayesha et al., 2025).

Based on this, this study aims to apply magnetic particle-based immunofluorescent technology—characterized by high sensitivity and specificity (Mariana et al., 2008)—to a self-developed microfluidic chip, designing and developing a multi-channel magnetic particle-based immunofluorescent disc-shaped microfluidic chip for the combined detection of antibodies against six common infectious diseases in pastoral areas (including visceral leishmaniasis, echinococcosis [cystic and alveolar echinococcosis], brucellosis, Lyme disease, and Xinjiang hemorrhagic fever). Specifically, the study will first conduct simulation tests on the chip’s fluid dynamics, then optimize the addition ratios of reagents and samples, followed by verification of performance indicators such as precision, accuracy, and specificity. Additionally, a consistency analysis will be performed between the detection results of this chip and those of the traditional enzyme-linked immunosorbent assay (ELISA).

The core value of this study lies in, on the one hand, relying on the characteristic that Xinjiang hemorrhagic fever virus (XHFV) IgM and *Borrelia* burgdorferi (Bb) IgM can be detected within 1–2 weeks of infection to achieve acute-phase screening of infectious diseases and accurately identify suspected cases such as the febrile phase of hantavirus infection and the erythema migrans phase of Lyme disease. On the other hand, it leverages the functions of IgG-class antibodies including rK39 IgG, XHFV IgG, and Em18 IgG to complete disease course confirmation and infection staging—for instance, the combined detection of XHFV IgG and IgM is used to determine whether a patient has entered the recovery phase (a decrease in IgM titer accompanied by an increase in IgG titer indicates improved condition), and a positive result for Em18 IgG suggests that echinococcosis has progressed to the advanced stage. Meanwhile, this study also utilizes the combined detection of lipopolysaccharide (LPS) antibodies and other antibodies to effectively distinguish between bacterial infections and parasitic infections, and correct misdiagnoses caused by cross-reactivity of a single antibody. For example, if XHFV IgM is positive while LPS antibodies are negative, it can rule out hemorrhagic fever-like symptoms caused by bacterial infections. In addition, the study can monitor the therapeutic effect of visceral leishmaniasis through dynamic changes in rK39 IgG titer, and follow up on the recurrence of echinococcosis patients after surgery based on the detection results of Em2 IgG. This multi-antibody combined detection mode is particularly suitable for scenarios with co-prevalence of multiple pathogens in pastoral areas, enabling one-time screening for common infections such as echinococcosis and Lyme disease. For difficult cases with atypical symptoms (e.g., fever of unknown origin), it can narrow down the diagnostic scope through antibody combination patterns. At the same time, detection items can be streamlined according to actual clinical needs (e.g., only XHFV IgM and Bb IgM are retained for early screening of acute febrile patients). While controlling detection costs, this study provides technical support for the timely diagnosis and efficient prevention and control of infectious diseases in pastoral areas, ultimately contributing to safeguarding the health of pastoral residents and the sustainable development of animal husbandry.

## 2 Materials and methods

### 2.1 Study subjects

From July 2024 to July 2025, for each detection item in this study, 60 serum samples positive for the corresponding antibody from human patients were enrolled. The serum samples included: 60 cases of *Leishmania* soluble antigen (SLA) IgG-positive serum from kala-azar patients, 60 cases of *Leishmania* recombinant K39 antigen (rK39) IgG-positive serum from kala-azar patients, 60 cases of *Echinococcus* granulosus antigen B (AgB) IgG-positive serum from cystic echinococcosis patients, 60 cases of *Echinococcus* granulosus antigen 5 (Ag5) IgG-positive serum from cystic echinococcosis patients, 60 cases of *Echinococcus* multilocularis antigen 2 (Em2) IgG-positive serum from alveolar echinococcosis patients, 60 cases of *Echinococcus* multilocularis antigen 18 (Em18) IgG-positive serum from alveolar echinococcosis patients, 60 cases of *Brucella* lipopolysaccharide (LPS) IgM-positive serum from brucellosis patients, 60 cases of *Brucella* lipopolysaccharide (LPS) IgG-positive serum from brucellosis patients, 60 cases of *Borrelia* burgdorferi (Bb) IgG-positive serum from Lyme disease patients, 60 cases of *Borrelia* burgdorferi (Bb) IgM-positive serum from Lyme disease patients, 60 cases of Xinjiang hemorrhagic fever virus (XHFV) IgG-positive serum, and 60 cases of Xinjiang hemorrhagic fever virus (XHFV) IgM-positive serum. All specimens were collected from the First Affiliated Hospital of Xinjiang Second Medical University, as well as the pastoral areas of Ili Kazakh Autonomous Prefecture, Tacheng Prefecture, and Altay Prefecture in Xinjiang.

The diagnostic criteria adopted in this study were as follows: the serological diagnostic criteria for visceral leishmaniasis (kala-azar) specified in Diagnostic Criteria for Kala-Azar (WS258-2006) (Ministry of Health of the People’s Republic of China, 2008), the serological diagnostic criteria for echinococcosis specified in Diagnostic Criteria for Echinococcosis (WS257-2006) (Ministry of Health of the People’s Republic of China, 2006), the serological diagnostic criteria for brucellosis specified in Diagnostic Criteria for Brucellosis (Note: the typo “WS269-20072” in the original text was corrected to “WS269-2007” in accordance with China’s official health standards) (Ministry of Health of the People’s Republic of China, 2007), the serological diagnostic criteria for Lyme disease specified in Diagnosis of Occupational Lyme Disease (GBZ324-2019) (National Health Commission of the People’s Republic of China, 2019), and the serological diagnostic criteria for hemorrhagic fever specified in Quarantine Technical Specification for Crimean-Congo Hemorrhagic Fever (SN/T5759-2024) (General Administration of Customs of the People’s Republic of China, 2024).

The inclusion criteria were specimens from individuals aged 18 to 80 years, with serum samples meeting the ELISA-positive criteria specified in the aforementioned documents. The exclusion criteria were specimens from individuals who had received anti-infective treatment within the past three months. This study has been approved by the Ethics Committee (Ethics Approval No.: MEC-XSMC-KT-20240630-001).

### 2.2 Instruments and reagents

#### 2.2.1 Instruments

HZZ-V3000 chip laser cutting machine (Huazhizun Optoelectronic Technology Co., Ltd., China), X350 microfluidic chip vacuum heat press (Xingweikong Biotechnology Co., Ltd., China), Olympus OLS5100 laser confocal fluorescence microscope (Olympus Corporation, Japan), Multiskan FC automated enzyme-linked immunosorbent assay reader (Thermo Fisher Scientific, USA), and H2D 3D printer (Bambu Lab Technology Co., Ltd., China).

#### 2.2.2 Reagents

Anti-human IgG monoclonal antibody (5 mg/mL) was purchased from Merck (USA); carboxyl magnetic beads (10 mg/mL) and carboxyl fluorescent microspheres (10 mg/mL) were obtained from Zhongke Leiming Co., Ltd. (China); polymethyl methacrylate (PMMA) chip plates were purchased from Trinseo (USA); ELISA detection kits for leishmaniasis and echinococcosis were sourced from Kerunda Bioengineering Co., Ltd. (China); the brucellosis ELISA detection kit was obtained from Harbin Guosheng Biotechnology Co., Ltd. (China); the Lyme disease ELISA detection kit was purchased from Shenzhen Ruiqing Bioinformation Technology Co., Ltd. (China); and the Crimean-Congo hemorrhagic fever ELISA detection kit was obtained from Xi’an Chuangyuan Biotechnology Co., Ltd. (China).

### 2.3 Methods

#### 2.3.1 The 2D and 3D design as well as processing and fabrication methods of microfluidic chips

**Figure 1:**
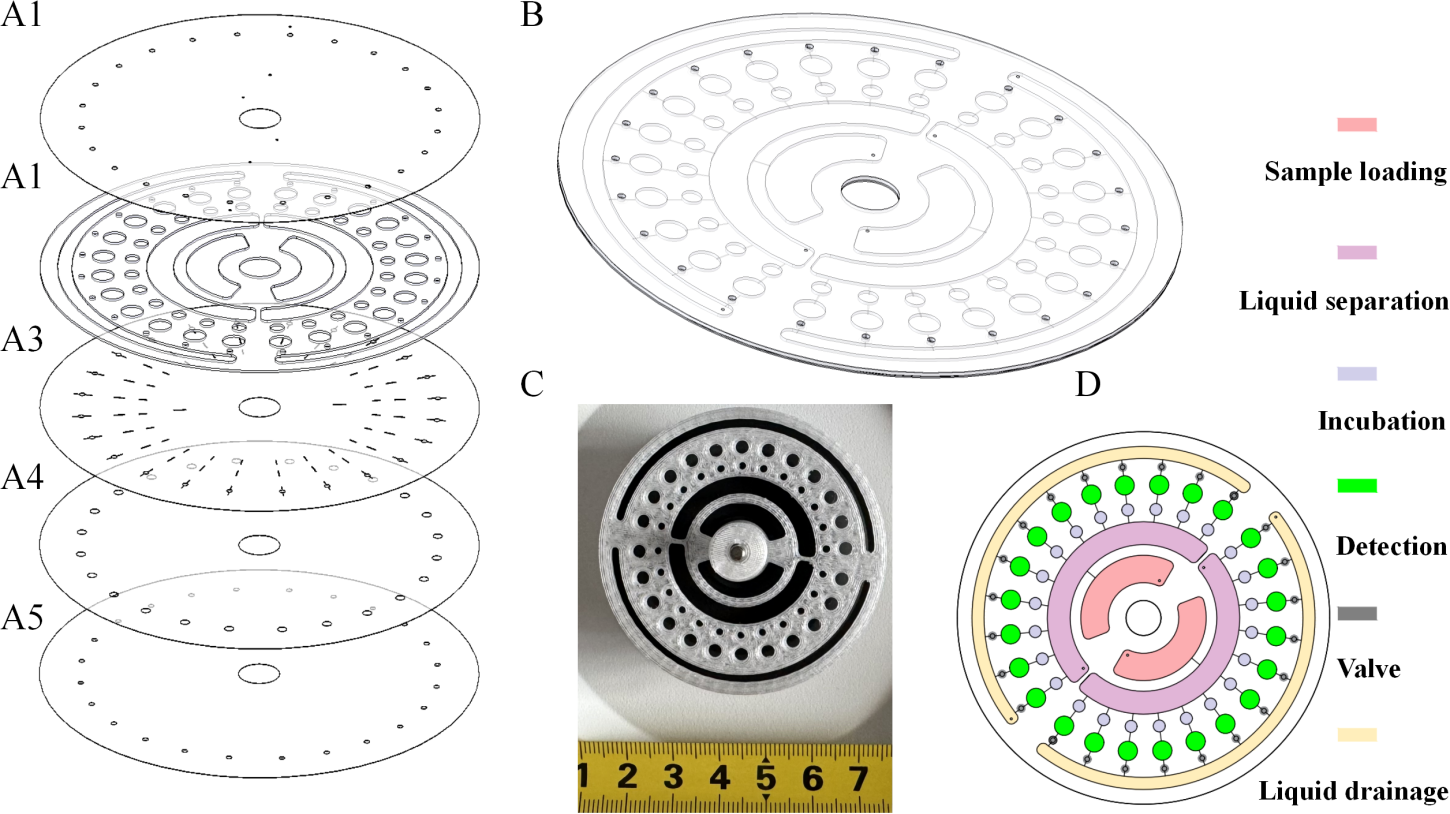
2D/3D structures and physical schematic diagrams of the chip. **(A1)** Constant pressure layer. **(A2)** Fluid chamber layer. **(A3)** Fluid channel layer. **(A4)** Fluid valve layer. **(A5)** Substrate layer. **(B)** Schematic diagram of the overall 3D structure of the chip. **(C)** Physical image of the fabricated chip. **(D)** Schematic diagram of the specific functions of each chamber.

First, the chip was designed and optimized using 2D and 3D software. Subsequently, its fabrication was completed via laser cutting and 3D printing technologies, and finally, its bonding and assembly were achieved using vacuum hot-pressing technology; details are shown in **Figure 1**.

This design serves as a core technical carrier for “multi-indicator combined detection” while supporting the performance of the magnetic particle immunofluorescence system, thereby laying a foundation for ensuring the accuracy of detection.

#### 2.3.2 Methods for conjugating antibodies and antigens to carboxyl-modified microspheres

In this study, chemical conjugation was employed to immobilize antigens and antibodies on carboxyl-modified microspheres. The specific procedure was as follows: carboxyl microspheres were first washed with MES buffer, then activated by adding 50 mM 1-ethyl-3-(3-dimethylaminopropyl)carbodiimide (EDC) for 15 minutes. Subsequently, antigens or antibodies were added, and the reaction was allowed to proceed for 16 hours. After the reaction, a 5% BSA solution was added for blocking, which lasted for 1 hour. Upon completion of blocking, the microspheres were washed three times with PBS buffer containing 0.1% Tween-20, yielding carboxyl microspheres conjugated with antigens or antibodies. This method serves as the “core connection hub” of the magnetic particle immunofluorescence detection system, ensuring the implementation of the detection principle.

#### 2.3.3 Experimental method for optimizing addition ratio

To achieve an optimal conjugation efficiency between antibodies/antigens and carboxyl microspheres, this study set up five groups with different mass ratios of microspheres to antibodies/antigens, specifically 10:1, 25:1, 50:1, 75:1, and 100:1. Each group was subjected to a stirring reaction for 3 hours under light-protected conditions. After blocking with BSA solution, the concentration of free antibodies in the supernatant was determined by ultraviolet spectrophotometry. The conjugation efficiency was calculated using the formula: “Conjugation efficiency (%) = (Total initial amount of antibodies - Amount of free antibodies in supernatant)/Total initial amount of antibodies × 100%”. Each ratio was measured in triplicate, and the mass ratio with the highest conjugation efficiency was finally selected as the optimal parameter for the reaction.

To achieve an optimal binding ratio between the sample and carboxyl fluorescent microspheres conjugated with anti-human IgG antibodies, this study serially diluted the same positive sample using a 2% BSA solution (containing 0.05% Tween-20) prepared in 0.01 mol/L PBS (pH 7.4), with four dilution ratios set as 1:50, 1:100, 1:200, and 1:400. Samples at each dilution were reacted with 1 μg, 5 μg, and 10 μg of activated fluorescent microspheres, respectively. After the reaction, the signal value and background value were measured, and the ratio of fluorescence intensity to background fluorescence was calculated. The condition corresponding to the maximum ratio was selected as the optimal parameter, and the entire experiment was repeated three times.

These experiments are designed to achieve the precise adaptation of each component in the detection system and ensure the feasibility of multi-indicator combined detection.

#### 2.3.4 Hydrodynamic simulation test method

The chip designed in this study features a multi-channel disc-shaped microfluidic structure, and its symmetric design ensures uniform liquid splitting. To verify whether it meets the requirements for fluid operation, this study conducted channel pressure tests using the hydrodynamic simulation software Comsol Multiphysics 6.3, while measuring the wall resolution to ensure the authenticity and reliability of the simulation results. The parameters for the hydrodynamic tests were set as follows: the serum density was set to 1020 kg/m^3^ with reference to the research results of Craft et al (Craft et al., 1993); the serum viscosity was set to 1.2 mPa·s based on the research data of Rosenson et al (Rosenson et al., 2002); the surface tension was determined as 48 mN/m according to the research conclusions of Rosina et al (Hrncír and Rosina, 1997); and the flow mode was set to laminar flow with reference to the research of Duffy et al (Duffy et al., 1999). The theoretical basis of the flow velocity calculation formula is derived from the combination of the Poiseuille flow principle and the radial pressure gradient generated by centrifugal force.

The key to centrifugal force-driven motion lies in the centrifugal acceleration generated by rotation.


$$\alpha =\omega^{2}r$$

Herein, ω represents the angular velocity (unit: radian per second), and the angular velocities set for this chip are 4 rad/s, 8 rad/s, 12 rad/s, and 16 rad/s in sequence; r denotes the rotational radius (unit: meter), and the rotational radii corresponding to the above four angular velocities are 0.08 m, 0.012 m, 0.016 m, and 0.024 m, respectively.

All fluid channels of this chip have a rectangular cross-section, and the calculation formula for the liquid flow velocity in such channels is as follows:


$$\upsilon =\frac{\rho h^{2}\alpha }{12\mu }$$

Herein, ρ denotes the fluid density (unit: kg/m^3^), which is a known parameter; h represents the channel height (unit: m) with a value of 0.0008 m; α stands for the centrifugal acceleration (unit: m/s^2^), which is obtained through calculation using Formula 1; and μ refers to the dynamic viscosity of the fluid (unit: Pa·s), which is also a known parameter.

After calculating the liquid flow velocity in the chip via the aforementioned formula, hydrodynamic simulation tests are conducted in accordance with the above parameters.

The experiments in this section are conducted to pre-verify the rationality of the chip’s fluid flow design and avoid the trial-and-error costs associated with the fabrication of physical microfluidic chips.

#### 2.3.5 Procedures, parameters and judgment criteria of the microfluidic chip

**Figure 2:**
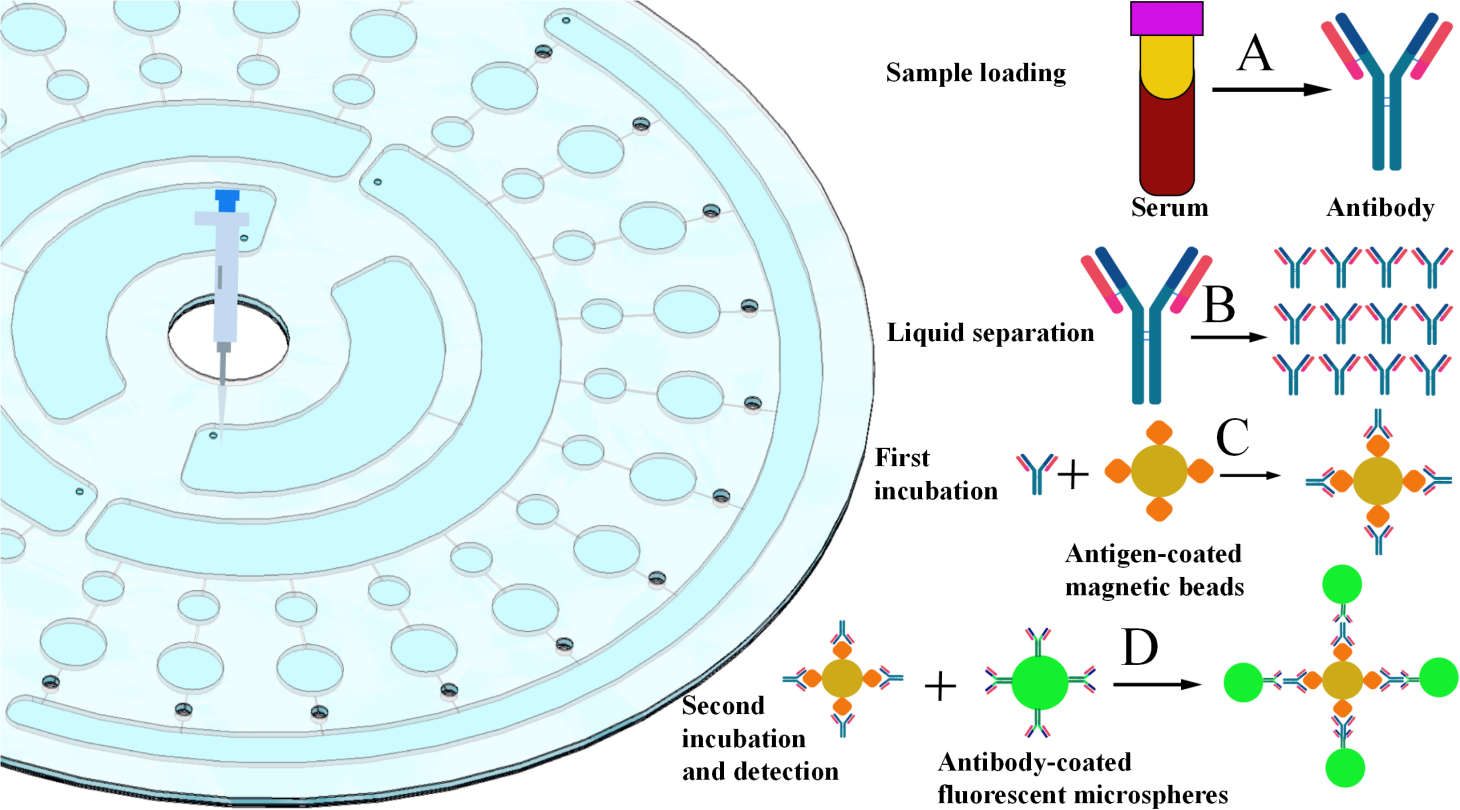
Detection process of the microfluidic chip. **(A)** Sample addition. **(B)** First-step reaction and washing. **(C)** Second-step reaction and washing. **(D)** Fluorescence detection.

The specific operating steps of the chip are as follows: First, the sample is added and centrifuged, after which it enters the antigen-magnetic bead storage chamber through the liquid separation pool. After the incubation and washing steps, the antibodies in the sample bind to the antigens to form antibody-antigen-magnetic bead complexes. Subsequently, centrifugation is performed again to transfer these complexes into the detection chamber. After another round of incubation and washing, the complexes bind to the secondary antibody-fluorescent microspheres, further forming magnetic bead-antigen-antibody-secondary antibody-fluorescent microsphere complexes. All excess waste liquid generated during the operation enters the waste liquid pool through centrifugation. Finally, fluorescence detection is carried out in the detection chamber, and the detection results of antibodies in the serum sample are obtained after data collation and analysis. The detailed operating process can be referred to in **Figure 2**.

In this study, FITC-labeled fluorescent microspheres were employed as the fluorescent signal reporters. The specific excitation/emission parameters and detection conditions for fluorescence signal acquisition were as follows: The fluorescence excitation wavelength was set to 488 nm, and the emission wavelength was 525 nm. Signal detection was performed using a fluorescence microscope with the following optimized parameters: excitation light intensity of 15%, exposure time of 150 ms, photomultiplier tube (PMT) gain of 80 dB, and a 10× fluorescent objective lens. A 520 ± 20 nm narrow-bandpass filter was applied to capture the specific fluorescent signals, which effectively eliminated background interference and guaranteed the stability, sensitivity, and reproducibility of the signal collection.

For multi-target synchronous detection, this study solely employed FITC-labeled fluorescent microspheres as the only fluorescence signal reporter; the identification of different pathogens was not realized by relying on the spectral differences of multiple fluorophores, but based on the spatial physical isolation of the microfluidic chip integrated with twelve independent and physically separated detection zones, each of which was modified with specific capture antibodies against the corresponding pathogens (immobilized on the fluorescent microspheres), so different analytes can be accurately identified and distinguished according to the spatial location of the fluorescent signals, which effectively avoids the spectral crosstalk caused by multi-fluorophore labeling and improves the accuracy and stability of detection.

Criteria for judgment are as follows: With the results of clinically commonly used ELISA detection as a reference, for the 12 items including SLA IgG, rK39 IgG, AgB IgG, Ag5 IgG, Em2 IgG, Em18 IgG, LPS IgM, LPS IgG, Bb IgG, Bb IgM, XHFV IgM, and XHFV IgG, the detection results of 60 positive samples and 60 negative samples were selected respectively. The optimal cut-off values for each item detected by this chip were determined through Receiver Operating Characteristic (ROC) curve analysis. Samples with detection values higher than the optimal cut-off value were judged as positive, and those lower than the optimal cut-off value were judged as negative.

The experiments in this section aim to lower the operational threshold to adapt to non-professional scenarios, match the characteristics of the disc-type chip to exert its structural advantages, and quantify the interpretation indicators to avoid subjective deviations.

#### 2.3.6 Experimental methods for verifying the performance of the chip

The process for reference material assignment and dose-response curve verification is as follows: The standard (100 μg/mL) of the ELISA kit was serially diluted using the kit’s accompanying diluent to prepare 6 serial reference materials with concentrations of 0 μg/mL (pure diluent), 6 μg/mL, 12 μg/mL, 24 μg/mL, 48 μg/mL, and 96 μg/mL, respectively. These serial reference materials were tested using the chip, with each concentration measured in triplicate to obtain the corresponding fluorescence values. The dose-response curve can be calculated based on the fluorescence values and the concentrations of the serial reference materials.

The determination method of the limit of detection is as follows: The standard with a concentration of 0 μg/mL is measured in 10 parallel experiments to obtain its fluorescence intensity values, and the mean and standard deviation of the results are calculated. The sum of the mean and twice the standard deviation is substituted into the standard curve equation, and the resulting value is the limit of detection.

The procedure for accuracy verification is as follows: Three types of samples are set up, including positive controls, negative controls, and blank controls. Among them, both positive controls and negative controls use standards from commercially available ELISA kits, while the blank control uses PBS buffer. These three types of control samples are tested separately, and the results under bright field and dark field are observed via a fluorescence microscope. Each sample is measured in triplicate to verify the accuracy of the chip detection.

The operational procedure for precision verification is as follows: High-value and low-value samples identified by clinical ELISA detection are selected, and each is subjected to 10 repeated detections using the microfluidic chip. The mean value and relative standard deviation of these 10 detection results are calculated, and the precision of the chip detection is verified through these two indicators.

The procedure for specificity verification is as follows: Five samples positive for each of the antibodies SLA IgG, rK39 IgG, AgB IgG, Ag5 IgG, Em2 IgG, Em18 IgG, LPS IgM, LPS IgG, Bb IgG, Bb IgM, XHFV IgM, and XHFV IgG are collected. These samples are individually tested using the present microfluidic chip, and the specificity of the chip detection is verified by observing whether cross-reactions occur.

The experiments in this section are intended to systematically verify the technical reliability of the microfluidic chip and connect with clinical needs, thereby providing direct evidence for the clinical applicability of the chip.

#### 2.3.7 Statistical analysis

Data processing and analysis were conducted using Microsoft Excel and GraphPad Prism 10 software. In line with the predefined inclusion and exclusion criteria, 12 types of serum biomarkers were tested, including SLA IgG, rK39 IgG, AgB IgG, Ag5 IgG, Em2 IgG, Em18 IgG, LPS IgM, LPS IgG, Bb IgG, Bb IgM, XHFV IgM, and XHFV IgG; for each biomarker, 60 positive serum samples and 60 negative serum samples were detected, amounting to a total of 1,440 samples. All samples were analyzed using two methods: the microfluidic chip-based antibody detection method and the ELISA antibody detection method, and each sample was tested in triplicate by both methods to reduce errors. The test data were first organized using Microsoft Excel and then imported into GraphPad Prism 10 software for statistical analysis; for consistency analysis between the two methods, a correlation plot was generated with the microfluidic chip detection data on the x-axis and the ELISA detection data on the y-axis, where a correlation coefficient (R^2^) greater than 0.95 indicated a good correlation between the two methods. To further verify the correlation, the Kappa test was used to analyze the consistency of the detection results, and a Kappa value ranging from 0.8 to 1.0 demonstrated good consistency of the detection results between the two methods; meanwhile, Bland-Altman consistency analysis was performed, and if more than 90% of the samples had detection results of the two methods falling within the 95% confidence interval, it also proved good consistency of the detection results between the two methods.

The experiments in this section are designed to align with the clinical “gold standard” and provide authoritative evidence for the clinical applicability of the microfluidic chip.

## 3 Results

### 3.1 Research results on the optimization of experimental addition ratios

**Table 1** presents the optimization results of the addition ratios of antigen-conjugated microspheres and antibody-conjugated microspheres, while **Table 2** shows the optimization results of the addition ratio of samples to microspheres. Experimental data indicate that the highest conjugation efficiency is achieved when the weight ratio of antibody-conjugated microspheres is 25:1 and the weight ratio of antigen-conjugated magnetic beads is 50:1. Additionally, the strongest specific signal intensity is obtained when the sample is diluted at a ratio of 1:200 and the microsphere dosage is 5 μg.

**Table 1:** Optimization results of antigen- or antibody-conjugated microspheres.

Range of weight ratio	Initial total amount of antibody (μg)	Amount of free antibody in the supernatant (μg)	Conjugation efficiency (antibody conjugated to microspheres) (%)	Initial total amount of antigen (μg)	Amount of free antigen in the supernatant (μg)	Conjugation efficiency (antigen conjugated to magnetic beads) (%)
10:1	10	3.31	66.9	10	3.59	64.1
25:1	10	1.22	87.8	10	2.51	74.9
50:1	10	1.28	87.2	10	1.31	86.9
75:1	10	1.77	82.3	10	2.19	78.1
100:1	10	2.69	73.1	10	2.64	73.6

**Table 2:** Optimization results of sample dilution ratios.

Dilution ratio	Amount of microspheres used (μg/mL)	Concentration converted from fluorescence intensity (μg/mL)	Concentration converted from background fluorescence (μg/mL)	Fluorescence intensity/Background fluorescence (%)
1:50	1	8.79	1.81	485.64
	5	17.96	3.36	534.52
	10	18.91	3.74	505.61
1:100	1	6.47	1.21	534.71
	5	16.03	2.54	631.10
	10	17.14	3.61	474.79
1:200	1	6.34	1.26	503.17
	5	13.46	1.34	1004.48
	10	13.88	1.78	779.78
1:400	1	6.05	1.03	587.38
	5	7.64	1.24	616.13
	10	8.69	1.93	450.26

Clinical scenarios have clear requirements for testing efficiency, sample volume, and operational complexity. Particularly, primary medical institutions in pastoral areas are confronted with practical challenges such as “limited sample volume, simple testing equipment, and urgent testing timelines”. The optimization results of additive ratios in this study can achieve in-depth adaptation to such scenarios.

### 3.2 Fluid dynamics test results

**Figure 3:**
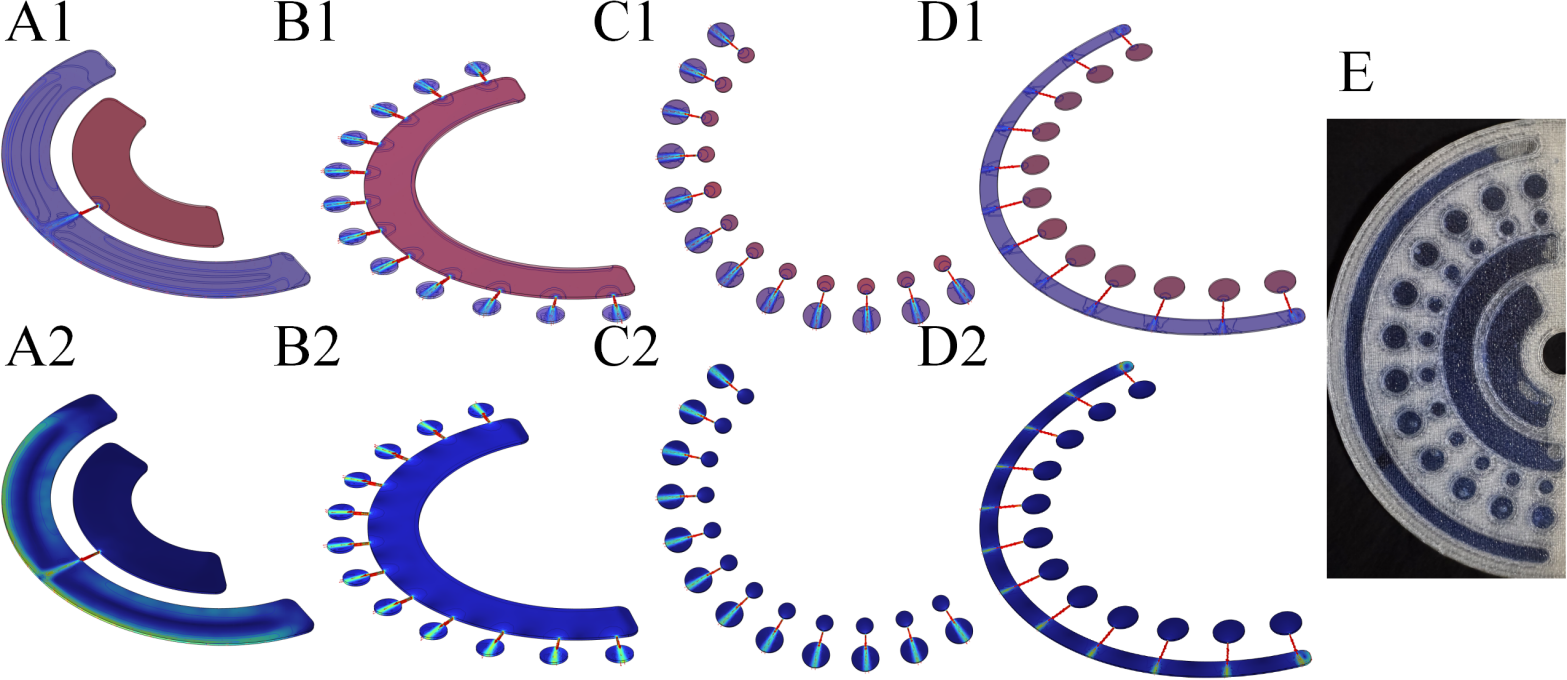
Diagram of chip fluid dynamics tests and physical fluid operation. **(A1)** Pressure test results of fluid flow from the sample loading pool to the distribution pool. **(A2)** Wall shear stress test results of fluid flow from the sample loading pool to the distribution pool. **(B1)** Pressure test results of fluid flow from the distribution pool to the first incubation pool. **(B2)** Wall shear stress test results of fluid flow from the distribution pool to the first incubation pool. **(C1)** Pressure test results of fluid flow from the first incubation pool to the second incubation pool. **(C2)** Wall shear stress test results of fluid flow from the first incubation pool to the second incubation pool. **(D1)** Pressure test results of fluid flow from the second incubation pool to the waste liquid pool. **(D2)** Wall shear stress test results of fluid flow from the second incubation pool to the waste liquid pool. **(E)** Physical test of fluid operation in the chip.

The test results of specific fluid dynamic pressure, wall shear stress in each chamber of the chip, and the test results of real liquid flow status are shown in **Figure 3**. The pressure test results of each chamber indicate that the pressure distribution of liquid flow in each chamber under the symmetrical structure is uniform, and the fluid operates normally. The wall shear stress test results of each chamber show that laminar flow can be maintained in each chamber and at the connecting structures, which ensures sufficient contact between the sample and the reaction bottom surface of the chip and improves the immune reaction efficiency of antigens and antibodies. The test results of real liquid flow status demonstrate that the actual fluid in each chamber operates normally.

### 3.3 Chip performance verification test

#### 3.3.1 Verification of chip detection time parameters and accuracy

The chip detection process consists of several key operational steps, with the time consumption of each step clearly quantified: The sample loading step takes an average of 1 minute, focusing on the precise transfer of the test sample to the pre-defined reaction region of the chip; The initial reaction phase lasts for an average of 5 minutes, whose core purpose is to achieve the specific binding between the target analyte and the specific probe. Upon completion of the reaction, a 2-minute washing operation is required to remove impurities with non-specific binding in the system; The secondary reaction consumes an average of 5 minutes, primarily enhancing the sensitivity of the detection system through a signal amplification mechanism. Subsequently, a 2-minute washing process is also performed to reduce background interference caused by non-specific signals; The detection operation phase takes an average of 1 minute to complete the collection of detection signals and preliminary data analysis.

Through cumulative calculation of the average time consumption of each step, the average total duration for a single chip detection is 16 minutes. Results of the time proportion analysis indicate that the cumulative time of the two reaction steps accounts for 62.5% (10/16) of the total detection time, the washing steps account for 25% (4/16), while the cumulative time of sample loading and detection operations only accounts for 12.5% (2/16). This suggests that the reaction process is the key link determining the time consumption of the entire detection process. From the perspective of application value, the 16-minute single detection cycle exhibits excellent temporal efficiency, which not only meets the practical requirements for detection speed in scenarios such as clinical rapid diagnosis and high-throughput screening of environmental samples but also effectively balances the specificity and accuracy of detection results through the process design of “two reactions and two washings”. This provides important time parameter support for the subsequent translational application and standardized promotion of the chip detection technology.

**Figure 4:**
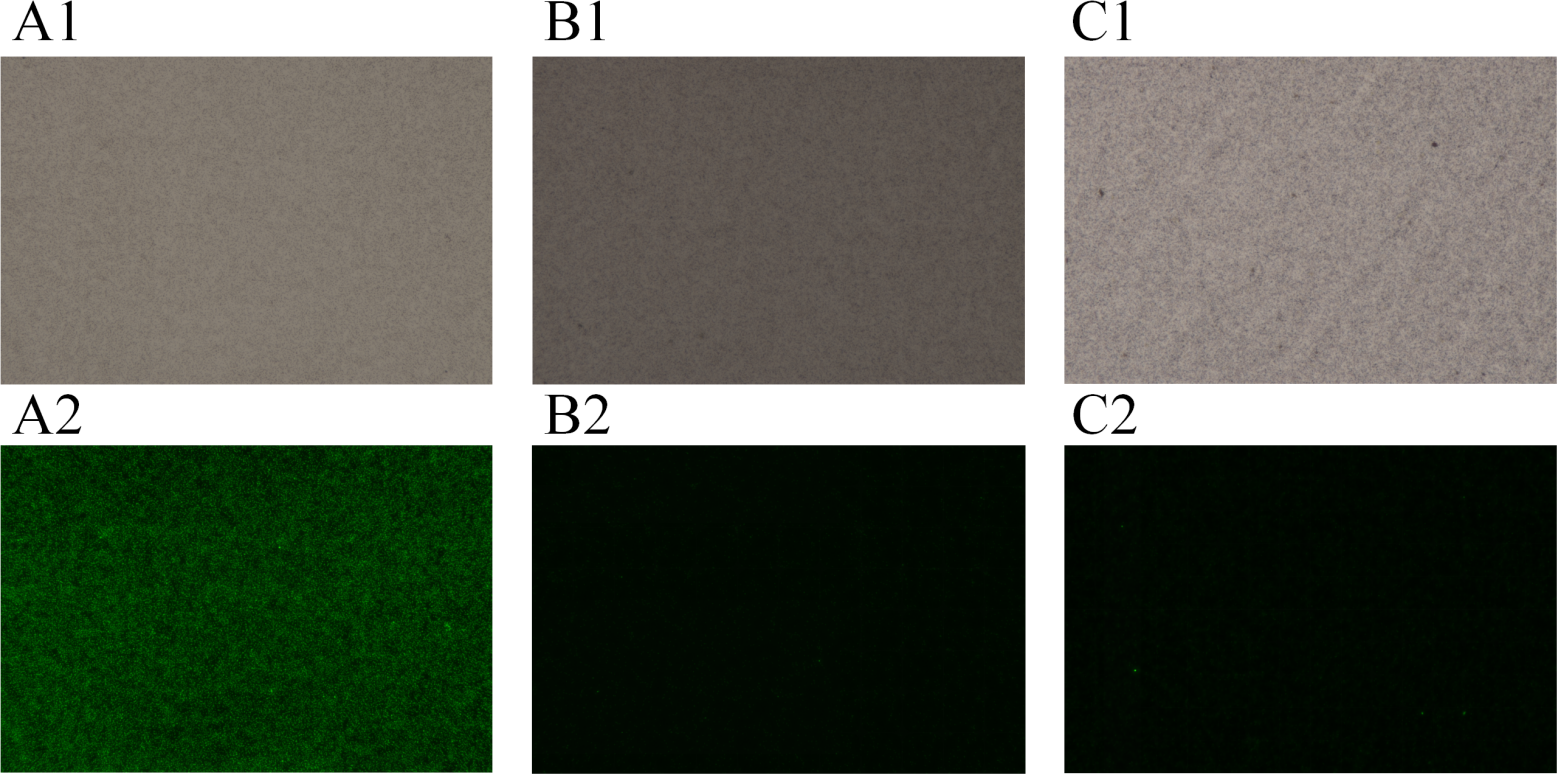
Performance verification result diagram of the microfluidic chip. **(A1)** Bright field of positive control; **(A2)** Dark field of positive control; (**B1)** Bright field of negative control; **(B2)** Dark field of negative control; **(C1)** Bright field of blank control; **(C2)** Dark field of blank control.

**Figure 4** shows the microscopic detection results of the positive control, negative control, and blank control under bright-field and dark-field conditions, respectively. The experiment was designed with three parallel replicates to ensure the statistical reliability of the data. From the dark-field imaging results, the positive control exhibited a clear and highly specific fluorescent signal, while no detectable fluorescent response was observed in the negative control and blank control. This phenomenon fully indicates that the detection system possesses excellent specificity, which can effectively eliminate false-positive results caused by non-specific fluorescent interference. Bright-field observation results revealed that magnetic bead adsorption was detectable in all three types of control samples, demonstrating that the targeted binding and enrichment process of magnetic beads in the chip reaction region is stable and controllable, with no occurrence of magnetic bead agglomeration or inconsistent adsorption efficiency.

The aforementioned results verified that the chip performance meets the established standards from two core performance dimensions: specificity and stability. The specific response characteristic—fluorescent signal present in the positive control but absent in the negative and blank controls—proves that the chip’s ability to recognize target analytes meets the experimentally set requirements. Meanwhile, the consistency of magnetic bead adsorption across the three types of controls further confirms that the repeatability and reliability of the chip reaction system comply with technical specifications. In summary, the chip has met the conditions for conducting subsequent target sample detection, methodological validation, and practical application scenario testing, laying an important technical foundation for the scientificity and accuracy of subsequent experimental data.

#### 3.3.2 Optimal cut-off value test

To comprehensively evaluate the detection performance of the chip, ROC curve analysis was performed separately for the 12 detection indicators integrated on the chip (SLA IgG, rK39 IgG, AgB IgG, Ag5 IgG, Em2 IgG, Em18 IgG, LPS IgM, LPS IgG, Bb IgG, Bb IgM, XHFV IgM, and XHFV IgG), aiming to clarify the key diagnostic efficacy parameters corresponding to each indicator. By calculating the sensitivity and specificity under different cut-off value conditions, the optimal cut-off value that balances diagnostic accuracy was selected, and quantitative analysis was conducted on the sensitivity, specificity, and area under the curve (AUC) of each indicator. All relevant data have been organized and summarized in **Table 3**.

**Table 3:** Table of ROC curve test results.

Item	Optimal cutoff value (μg/mL)	Sensitivity (%)	Specificity (%)	Area under the curve
AgB IgG	1.17	96	100	0.998
Ag5 IgG	0.96	98.3	100	0.999
Em2 IgG	0.8	94.9	100	0.996
Em18 IgG	0.91	91.5	100	0.998
LPS IgG	0.91	93.2	100	0.998
LPS IgM	0.8	96.6	100	0.998
Bb IgM	0.74	96.6	100	0.998
Bb IgG	0.73	96.6	98.4	0.998
rK39 IgG	0.94	96.6	100	0.998
SLA IgG	1.07	94.8	100	0.997
XHFV IgM	1.16	96.6	98.4	0.998
XHFV IgG	0.74	93.2	100	0.999

The results of ROC curve analysis indicated that the AUC values of all 12 detection indicators were no less than 0.95, which confirms that the chip possesses excellent diagnostic discrimination efficacy for target antibodies/antigens. Meanwhile, the sensitivity and specificity of all indicators exceeded 90%, demonstrating that the chip not only has high detection capability in clinical sample testing but also can effectively control the risk of misjudgment, enabling accurate differentiation between positive and negative samples. The determination of the optimal cut-off value for each indicator provides a core quantitative reference for the standardized interpretation of subsequent chip detection processes, which helps ensure the objectivity and consistency of detection results across different batches and laboratories.

#### 3.3.3 Dose-response curve and limit of detection test

**Figure 5:**
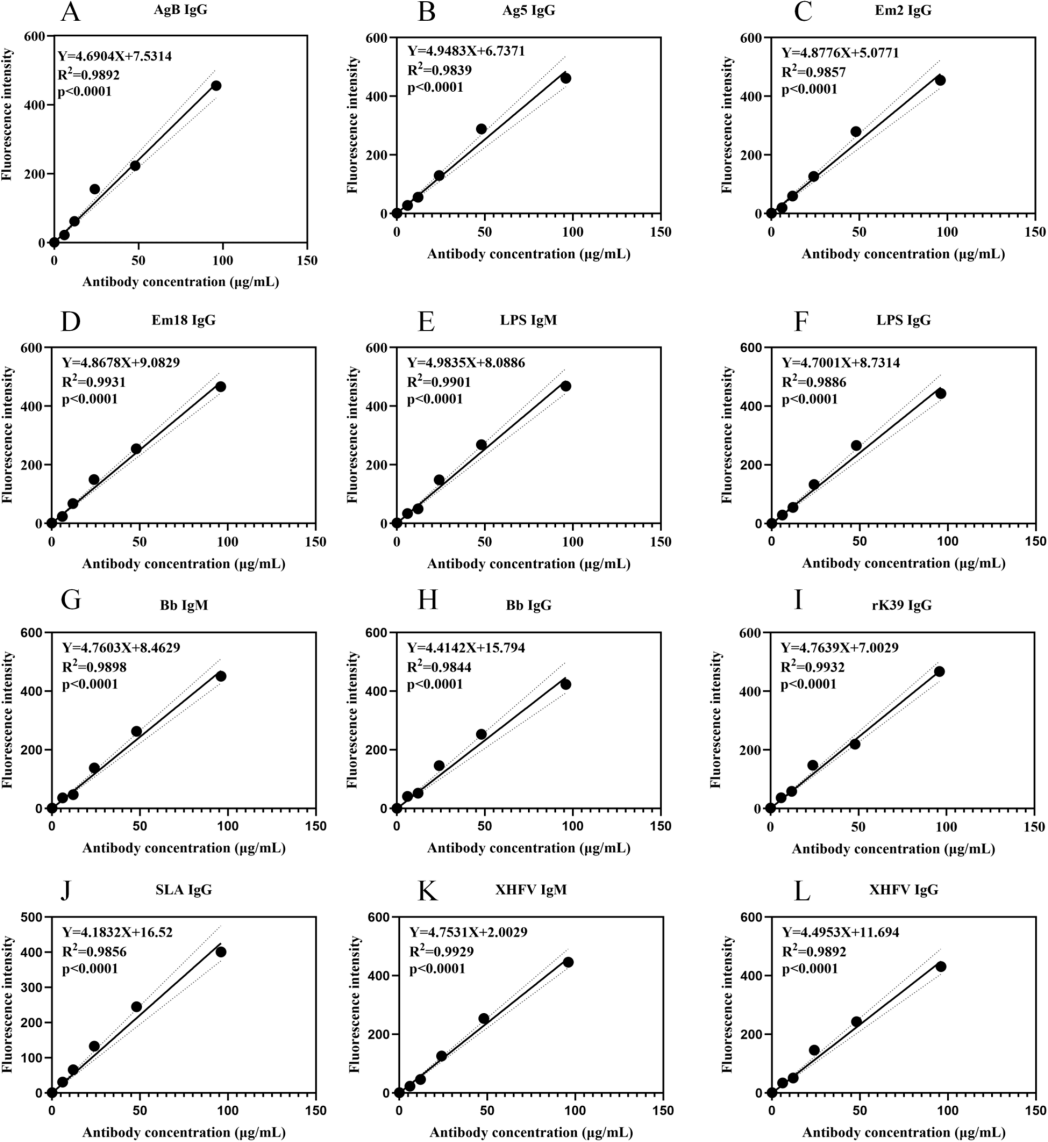
Calibration curves of the 12 detection items. **(A)** represents the calibration curve of AgB IgG, **(B)** represents that of Ag5 IgG, **(C)** represents that of Em2 IgG, **(D)** represents that of Em18 IgG, **(E)** represents that of LPS IgM, **(F)** represents that of LPS IgG, **(G)** represents that of Bb IgM, **(H)** represents that of Bb IgG, **(I)** represents that of rK39 IgG, **(J)** represents that of SLA IgG, **(K)** represents that of XHFV IgM, and **(L)** represents that of XHFV IgG.

After systematic data processing and statistical analysis, the core performance parameters of each detection item—including the coefficient of determination (R^2^) of the linear regression equation, linear range, and limit of detection (LOD)—have been organized and summarized in **Table 4**, and the calibration curves corresponding to the 12 detection items are shown in **Figure 5**. The results confirm that the dose-response curves of the chip for each target analyte exhibit excellent linear fitting effects, with the R^2^ values of all detection items being no less than 0.98 (see **Table 4** for specific parameters). This indicates a significant correlation between the chip’s detection signal and the target analyte concentration, providing strong support for the reliability of quantitative analysis. Meanwhile, the linear range of the chip can fully cover the conventional concentration intervals of target analytes in clinical samples and practical detection scenarios, enabling accurate quantitative analysis without the need for additional sample dilution or concentration. Its LOD values all meet the preset design standards and are lower than the minimum detection threshold of target analytes in practical applications, allowing for highly sensitive detection of low-concentration samples.

**Table 4:** Table of dose-response curve and limit of detection test results.

Item	Dose-response curve equation	*R* ^2^	Linear range (μg/mL)	Limit of detection (μg/mL)
AgB IgG	Y=4.6904X+7.5314	0.9892	0.6-96	0.6
Ag5 IgG	Y=4.9483X+6.7371	0.9839	0.8-96	0.8
Em2 IgG	Y=4.8776X+5.0771	0.9857	0.7-96	0.7
Em18 IgG	Y=4.8678X+9.0829	0.9931	0.9-96	0.9
LPS IgG	Y=4.7001X+8.7314	0.9886	0.6-96	0.6
LPS IgM	Y=4.9385X+8.0886	0.9901	1.1-96	1.1
Bb IgM	Y=4.7603X+8.4629	0.9898	1.2-96	1.2
Bb IgG	Y=4.4142X+15.794	0.9844	0.8-96	0.8
rK39 IgG	Y=4.7639X+7.0029	0.9932	1.1-96	1.1
SLA IgG	Y=4.1832X+16.52	0.9856	1.2-96	1.2
XHFV IgM	Y=4.7531X+2.0029	0.9929	1.1-96	1.1
XHFV IgG	Y=4.4953X+11.694	0.9892	1.3-96	1.3

The aforementioned results comprehensively verify the chip’s comprehensive performance from three core dimensions: quantitative reliability, practical applicability, and detection sensitivity. High R^2^ values ensure the stability and credibility of quantitative analysis results; the wide linear range enhances the chip’s adaptability to samples of different concentrations and expands its application scenarios; and the low LOD values guarantee the effective capture and detection of low-concentration target analytes. In summary, the chip performs outstandingly in key performance indicators such as quantitative accuracy, concentration adaptability range, and detection sensitivity, which can fully meet the strict requirements of subsequent in-depth experimental research, methodological validation, and practical detection applications of clinical or environmental samples. This lays an important technical foundation for the further translational implementation and large-scale promotion of the technology.

#### 3.3.4 Repeatability test

Relevant test data regarding the detection repeatability of the chip are detailed in **Table 5**. In this study, a comprehensive evaluation of the chip’s detection repeatability was conducted through systematic experiments. The results show that the standard deviations (SD) of all repeated detection results fall within the preset allowable range. This fully confirms that the detection repeatability of the chip has completely met the technical standard requirements set during the design phase, and the stability and reliability of its detection system have been fully verified.

**Table 5:** Repeatability test results.

Analytical items	Sample types	Mean value (μg/mL)	Relative standard deviation (%)
AgB IgG	Low value	14.85	2.14
	High value	61.63	2.6
Ag5 IgG	Low value	10.24	5.05
	High value	75.92	1.22
Em2 IgG	Low value	12.91	3.51
	High value	66.33	1.35
Em18 IgG	Low value	16.84	2.35
	High value	87.48	1.36
LPS IgG	Low value	22.24	3.68
	High value	77.58	1.16
LPS IgM	Low value	19.50	4.56
	High value	62.47	1.4
Bb IgM	Low value	24.18	1.92
	High value	71.94	1.01
Bb IgG	Low value	15.1	4.14
	High value	77.16	1.3
rK39 IgG	Low value	22.18	5.26
	High value	61.79	1.54
SLA IgG	Low value	19.68	4.04
	High value	58.44	2.51
XHFV IgM	Low value	12.26	6.44
	High value	72.16	2.46
XHFV IgG	Low value	19.69	4.6
	High value	57.49	2.94

Excellent detection repeatability can not only effectively reduce the interference of random errors in experimental operations on detection results, ensuring the repeatability and horizontal comparability of detection data across different batches and time points, but also provide stable and reliable technical support for the standardized advancement of subsequent experimental research and the efficient analysis of batch samples in practical detection scenarios. This further confirms that the chip has acquired the core performance conditions for large-scale promotion and application.

#### 3.3.5 Specificity test

To comprehensively verify the detection specificity of the chip, this study conducted a systematic specificity evaluation using the cross-reactivity validation method for the 12 detection indicators integrated on the chip (AgB IgG, Ag5 IgG, Em2 IgG, Em18 IgG, rK39 IgG, SLA IgG, LPS IgM, LPS IgG, Bb IgG, Bb IgM, XHFV IgM, and XHFV IgG). The experimental results demonstrated that all detection indicators exclusively bind specifically to their corresponding targets, without generating any cross-reactivity signals with the targets of the other 11 indicators, which fully reflects the target specificity of each indicator’s recognition system.

This result strongly confirms that the specificity of the chip has fully met the preset design standards. The probes for each detection indicator equipped on the chip possess excellent target recognition specificity, enabling effective avoidance of cross-interference between different indicators. As one of the core performances guaranteeing the reliability of detection results, superior specificity can fundamentally eliminate false-positive or false-negative misjudgments caused by cross-reactivity. It provides key technical support for subsequent multi-indicator simultaneous detection of complex samples, methodological validation, and accurate detection in practical application scenarios, further verifying the core performance advantages of the chip in clinical translation and large-scale promotion and application.

### 3.4 Consistency analysis with the ELISA method

**Figure 6:**
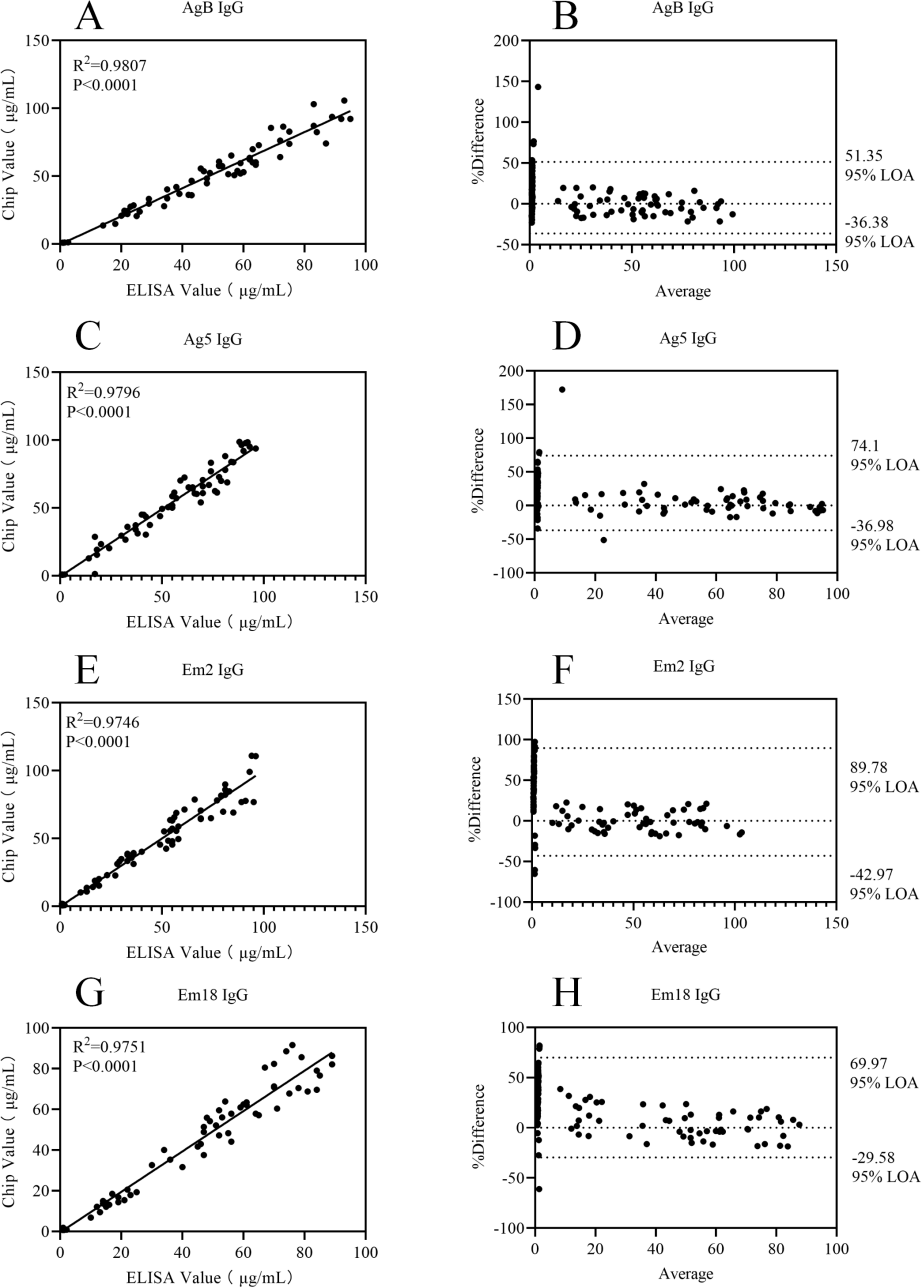
Correlation scatter plots and Bland-Altman plots comparing ELISA and Chip assay results for AgB IgG, Ag5 IgG, Em2 IgG, and Em18 IgG detection assays. **(A)** Correlation scatter plot of ELISA and Chip assay results for AgB IgG; **(B)** Bland-Altman plot of ELISA and Chip assay results for AgB IgG; **(C)** Correlation scatter plot of ELISA and Chip assay results for Ag5 IgG; **(D)** Bland-Altman plot of ELISA and Chip assay results for Ag5 IgG; **(E)** Correlation scatter plot of ELISA and Chip assay results for Em2 IgG; **(F)** Bland-Altman plot of ELISA and Chip assay results for Em2 IgG; **(G)** Correlation scatter plot of ELISA and Chip assay results for Em18 IgG; **(H)** Bland-Altman plot of ELISA and Chip assay results for Em18 IgG.

**Figure 7:**
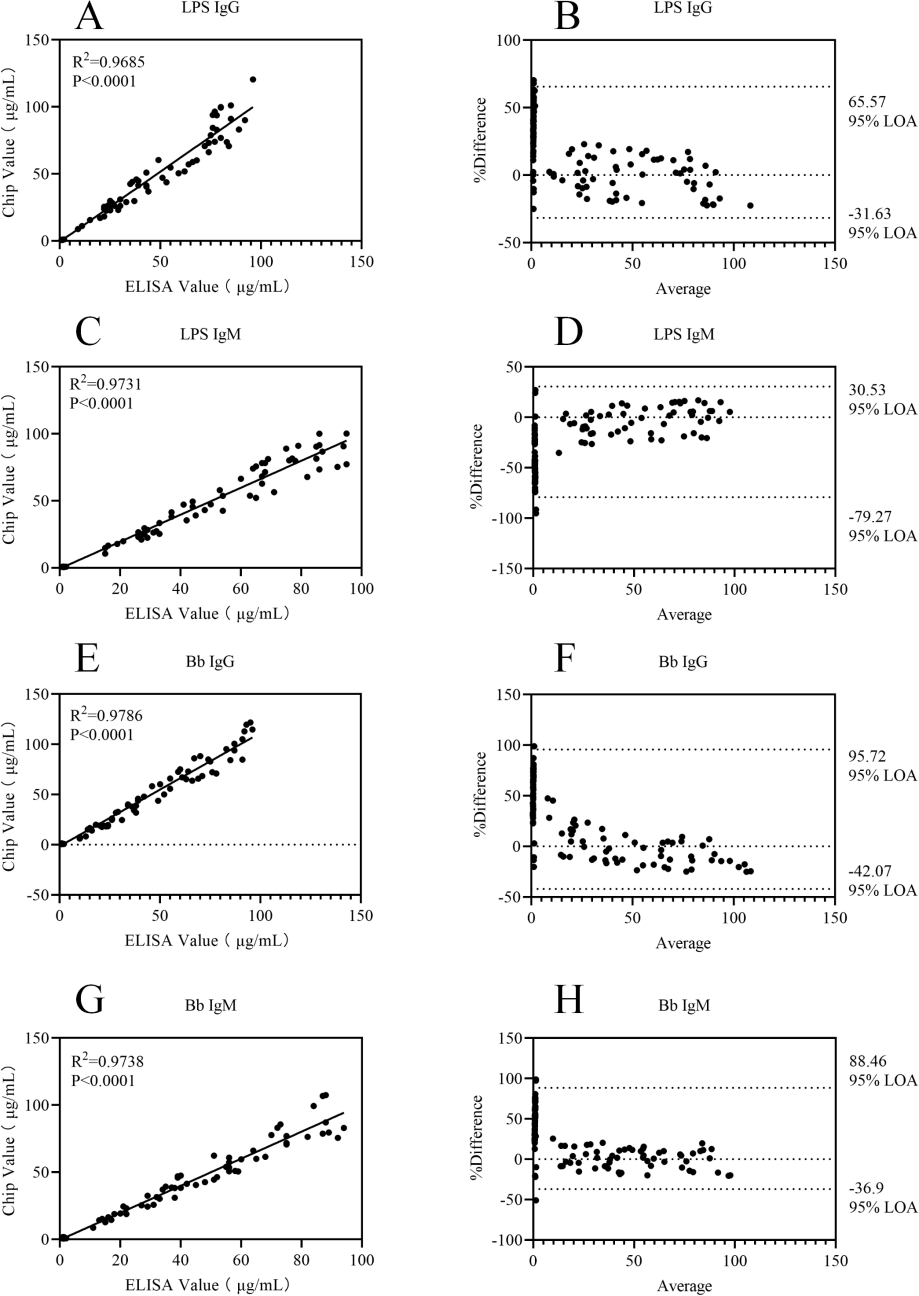
Correlation scatter plots and Bland-Altman plots comparing ELISA and Chip assay results for LPS IgG, LPS IgM, Bb IgG, and Bb IgM detection assays. **(A)** Correlation scatter plot of ELISA and Chip assay results for LPS IgG; **(B)** Bland-Altman plot of ELISA and Chip assay results for LPS IgG; **(C)** Correlation scatter plot of ELISA and Chip assay results for LPS IgM; **(D)** Bland-Altman plot of ELISA and Chip assay results for LPS IgM; **(E)** Correlation scatter plot of ELISA and Chip assay results for Bb IgG; **(F)** Bland-Altman plot of ELISA and Chip assay results for Bb IgG; **(G)** Correlation scatter plot of ELISA and Chip assay results for Bb IgM; **(H)** Bland-Altman plot of ELISA and Chip assay results for Bb IgM.

**Figure 8:**
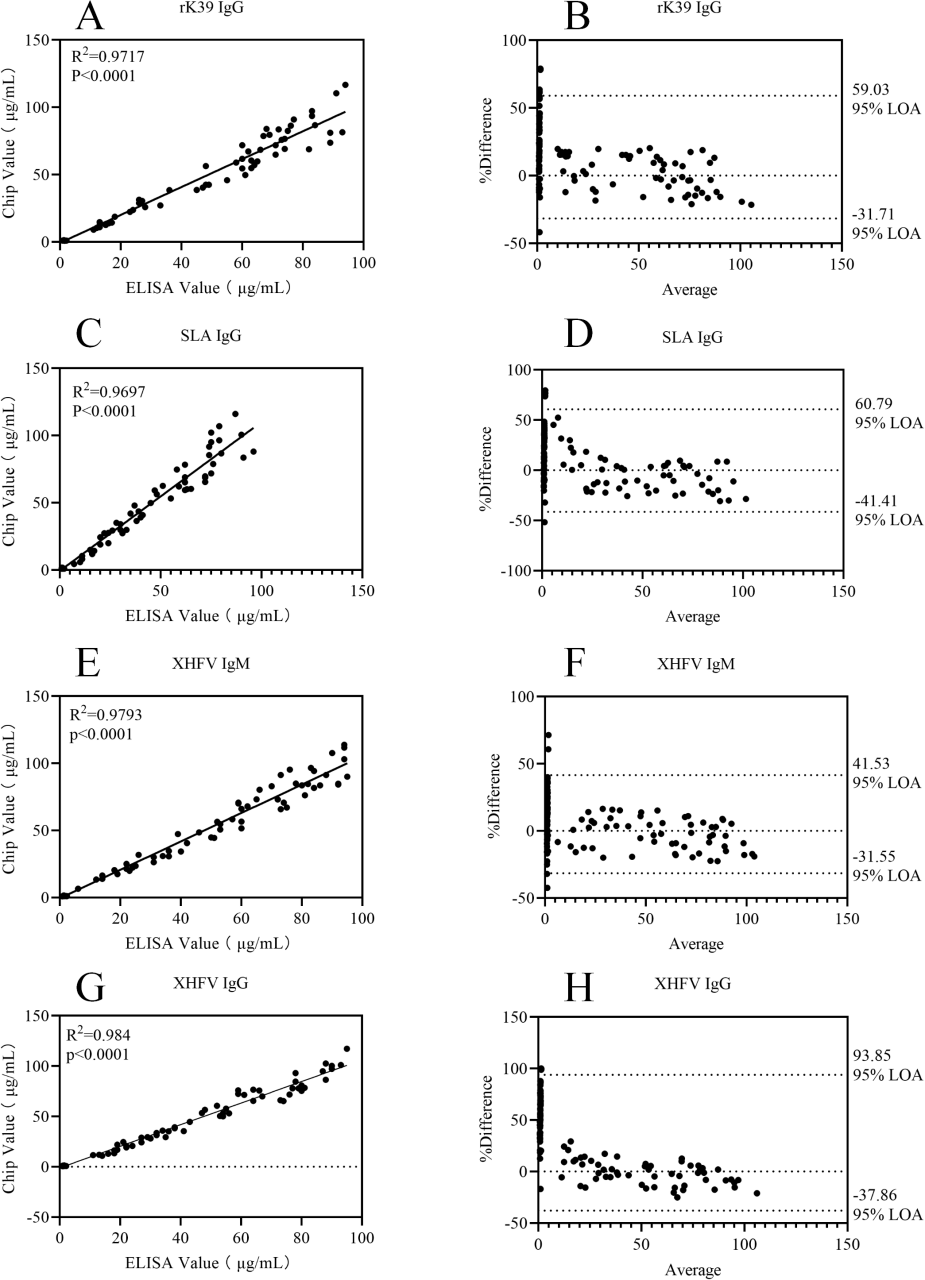
Correlation scatter plots and Bland-Altman plots comparing ELISA and Chip assay results for rK39 IgG, SLA IgG, XHFV IgM, and XHFV IgG detection assays. **(A)** Correlation scatter plot of ELISA and Chip assay results for rK39 IgG; **(B)** Bland-Altman plot of ELISA and Chip assay results for rK39 IgG; **(C)** Correlation scatter plot of ELISA and Chip assay results for SLA IgG; **(D)** Bland-Altman plot of ELISA and Chip assay results for SLA IgG; **(E)** Correlation scatter plot of ELISA and Chip assay results for XHFV IgM; **(F)** Bland-Altman plot of ELISA and Chip assay results for XHFV IgM; **(G)** Correlation scatter plot of ELISA and Chip assay results for XHFV IgG; **(H)** Bland-Altman plot of ELISA and Chip assay results for XHFV IgG.

The results of statistical analysis showed that for each of the 12 detection indicators, the coefficient of determination (R^2^) in the correlation scatter plots of the two detection methods was greater than 0.95, as detailed in **Figures 6**–**8**. The results of the Kappa test indicated that all Kappa values ranged from 0.8 to 1.0 (see **Table 6** for specific data). For the Bland-Altman analysis, the proportion of samples whose detection results fell within the 95% confidence interval (95% CI) exceeded 90% for all indicators, and the deviations within this interval were all within the clinically acceptable error threshold.

**Table 6:** Table of Kappa test results.

Item	Coincident rate		Kappa
	Positive	Negative	
AgB IgG	96.67% (58/60)	93.33% (56/60)	0.9
Ag5 IgG	95% (57/60)	91.67% (55/60)	0.867
Em2 IgG	93.33% (56/60)	91.67% (55/60)	0.85
Em18 IgG	95% (57/60)	95% (57/60)	0.9
LPS IgG	96.67% (58/60)	95% (57/60)	0.917
LPS IgM	96.67% (58/60)	95% (57/60)	0.917
Bb IgM	96.67% (58/60)	91.67% (55/60)	0.883
Bb IgG	98.33% (59/60)	93.33% (56/60)	0.917
rK39 IgG	96.67% (58/60)	93.33% (56/60)	0.9
SLA IgG	93.33% (56/60)	95% (57/60)	0.883
XHFV IgM	96.67% (58/60)	95% (57/60)	0.917
XHFV IgG	96.67% (58/60)	95% (57/60)	0.917

These analysis results fully confirm that the detection results of the microfluidic chip are highly consistent with those of ELISA, one of the clinical “gold standards”. This conclusion not only provides key data support for the microfluidic chip to replace or supplement ELISA in clinical sample detection, but also lays a solid foundation for the subsequent promotion and popularization of its clinical application.

## 4 Discussion

The multi-channel disc-shaped magnetic particle immunofluorescence microfluidic chip designed in this study takes meeting the needs of acute-phase screening, disease course confirmation, infection staging, differentiation between bacterial and parasitic infections, and disease prognosis detection for multiple prevalent infectious diseases in pastoral areas as its core development objective.

Compared with the clinically used enzyme-linked immunosorbent assay (ELISA), this chip offers greater advantages in application scenarios and comprehensive benefits. In terms of on-site adaptability, traditional ELISA relies on laboratory settings, whereas this chip features portability and on-site detection capability. For mixed infection detection, traditional ELISA cannot simultaneously detect multiple disease markers, while this chip enables multi-marker detection. In terms of single-test cost, the cost of this chip is roughly equivalent to that of traditional ELISA; however, since the chip can detect more markers, it reduces the probability of missed diagnosis of other diseases in patients to a certain extent, thereby lowering patients’ medical costs.

Compared with existing centrifugal microfluidic technologies, this chip has greater advantages in detection throughput and application objectives. Although existing centrifugal microfluidic technologies have achieved preliminary parallelization in multi-pathogen detection, there remain shortcomings in the compatibility between throughput and efficiency. For instance, the integrated centrifugal microfluidic chip developed by He et al. can simultaneously detect 6 types of bacteria, but the total detection time is as long as 70 minutes, and it only covers bacterial targets. Designed for “laboratory-accurate detection,” this chip cannot address the mixed prevalence of “parasites-bacteria-viruses” in special scenarios such as pastoral areas. Additionally, it relies on PCR technology, requiring nucleic acid extraction from samples, which results in high operational complexity and difficulty for primary-level personnel to master (Yan et al., 2017). The microfluidic chip developed by Goral et al. achieves automated nucleic acid detection, but its detection targets are strictly limited to a few bacterial species (e.g., Staphylococcus and Streptococcus), and each test requires the design of PCR primers for a single bacterial species. Thus, it cannot simultaneously detect multiple markers or meet the requirement for multi-marker combined detection in special scenarios (Czilwik et al., 2015).

The core breakthroughs of this study lie in the coordinated design of precise multi-antibody parallel detection and scenario-driven objective reconstruction: ① For the first time, a combined detection system for “12 antibodies against 6 zoonotic diseases” was established, covering pastoral area-specific diseases such as visceral leishmaniasis and Xinjiang hemorrhagic fever; ② By optimizing the centrifugal flow channel distribution structure of the disc chip, 12 characteristic antibodies of 6 high-incidence zoonotic diseases in pastoral areas were integrated into the simultaneous detection system, resulting in a higher number of detection channels; ③ Adopting a centrifugal-driven mode with simple operation, the chip integrates sample addition, reaction, washing, and detection modules onto a single disc, eliminating the need for complex external equipment such as pumps and valves. Primary-level personnel in local areas can master the detection technology after only 1 hour of training; ④ Integrating magnetic particle-based immunofluorescence technology, the chip uses microspheres to efficiently capture target antibodies, achieving high sensitivity. Moreover, the fluorescent signal is less affected by temperature fluctuations than enzymatic reactions, making it suitable for the extreme temperature range of -20~40°C in pastoral areas. More importantly, relying on the rapid reaction characteristics of magnetic particle-based immunofluorescence technology (antigen-antibody binding time shortened to 8 minutes), the entire detection process can be completed within 16 minutes, significantly improving detection efficiency. These features perfectly match the clinical pain points of “mixed prevalence of multiple pathogens and overlapping symptoms” in pastoral areas.

From a clinical practice perspective, through the “combined detection of antibodies against 6 diseases,” the chip can quickly provide more accurate guidance for clinical treatment. For example, if a patient presents with fever and hepatosplenomegaly, and the chip detection results show “visceral leishmaniasis IgG positive and the other 5 antibodies negative,” it strongly suggests that the patient may suffer from visceral leishmaniasis, providing a basis for clinical treatment. Co-infection cases of brucellosis and echinococcosis are also common in pastoral areas, and the chip can identify such cases as “brucellosis IgM positive + echinococcosis IgG positive.” This more accurate detection result of co-infection enables clinicians to formulate treatment plans with greater confidence (e.g., brucellosis requires combined antibiotic therapy, while echinococcosis requires surgery combined with anti-parasitic drugs). In addition, the chip’s rapid detection capability avoids the problem of patients “making multiple trips to hospitals and waiting for results for a long time” (the average one-way travel time for medical treatment in pastoral areas is 1–2 hours), thereby reducing the risk of adverse symptoms in patients due to prolonged waiting.

Despite the satisfactory performance of the microfluidic chip developed in this study in terms of integration, automation, detection sensitivity and specificity, several limitations still need to be further optimized. Non-standardized sample collection in pastoral areas tends to cause sample hemolysis, and massive hemoglobin in hemolyzed samples may interfere with the detection results, so a hemoglobin adsorption module will be further designed and integrated into the chip in subsequent research to reduce the interference of hemolyzed samples and enhance the application value of the chip in pastoral areas. In addition, the cost of the chip and supporting reagents can be further optimized: large-scale production can be adopted to reduce the chip cost, and the independent preparation of commercial magnetic beads and microspheres will be explored to further lower the overall detection cost. In this study, a fluorescence microscope was used for signal collection and quantitative analysis in the laboratory validation stage to realize the accurate and reliable evaluation of the chip’s analytical performance, and to further promote its practical application in resource-limited pastoral areas, the development of a portable integrated detection device will be the focus of our future research, specifically, a miniaturized battery-powered signal reader combined with a smartphone application will be designed and developed to achieve on-site, rapid and instrument-independent detection of multiple infectious diseases in pastoral areas.

In conclusion, this chip provides a novel detection technology for the prevention and control of infectious diseases in pastoral areas. It is expected to contribute to the achievement of the prevention and control goals of “early detection, early intervention, and mortality reduction” in pastoral areas, and offer support for the accurate diagnosis and treatment of infectious diseases there. Therefore, it holds broad application prospects in pastoral areas affected by infectious disease outbreaks.

## Data Availability

The original contributions presented in the study are included in the article/supplementary material. Further inquiries can be directed to the corresponding author.
